# The Global Epidemiology of Antimicrobial Resistance: Trends, Determinants, and Public Health Implications

**DOI:** 10.7759/cureus.100784

**Published:** 2026-01-04

**Authors:** Saud Lafi Oudah Alenzi, Ali Hedayan Alharthi, Raad Awwadh Alnefaie, Abdulaziz Alzahrani, Osama Alsaadi, Abdulaziz Naif Mohammed Albugami, Khalid Mohammed Alhusini, Foz Salem Alanazi, Fahad Hamdi Khalif ALAnazi, Alaa Abduljabber Alkhayyat

**Affiliations:** 1 Department of Pharmaceutical Care, Ministry of National Guard Health Affairs, Jeddah, SAU; 2 Department of Medical Committee, Ministry of National Guard Health Affairs, Jeddah, SAU; 3 Department of Medical Laboratory, Ministry of National Guard Health Affairs, Jeddah, SAU; 4 Department of Pathology and Laboratory Medicine, Ministry of National Guard Health Affairs, Taif, SAU; 5 Department of Cardiac Cath Lab, Ministry of National Guard Health Affairs, Riyadh, SAU; 6 Department of Nursing, Ministry of National Guard Health Affairs, Riyadh, SAU; 7 Department of Healthcare Technology Management, Ministry of National Guard Health Affairs, Jeddah, SAU

**Keywords:** : antimicrobial resistance, epidemiology, global health, one health, public health policy

## Abstract

Antimicrobial resistance (AMR) represents one of the most pressing global health and development challenges of the twenty-first century, threatening the effectiveness of antimicrobial therapies and undermining the clinical management of infectious diseases worldwide. The increasing prevalence of resistant pathogens undermines progress in disease control, surgery, and critical care, and poses severe socioeconomic and public health consequences. This narrative review synthesizes multidisciplinary evidence to examine the global trends, determinants, and implications of AMR through an integrated One Health framework encompassing human, animal, and environmental systems. The review draws upon peer-reviewed literature, international surveillance data, and policy reports published between 2015 and 2025 to provide a comprehensive understanding of resistance patterns and drivers. Findings reveal that resistance arises from an interplay of inappropriate antimicrobial use, insufficient infection prevention, environmental contamination, and weak governance, with the heaviest burden in low- and middle-income countries. Despite global action plans and stewardship initiatives, progress remains constrained by inequitable resources, limited diagnostics, and fragmented implementation. Strengthening surveillance systems, fostering responsible antimicrobial use, promoting research and vaccine development, and embedding One Health principles in policy are vital to reversing current trends. Coordinated global action, equitable investment, and sustained political commitment are essential to preserve antimicrobial efficacy and ensure the resilience of health systems worldwide.

## Introduction and background

Antimicrobial resistance (AMR) is one of the biggest threats to global health and development of the twenty-first century, undermining the effectiveness of antimicrobial therapies and endangering global health security [1]. Antimicrobials, once hailed as revolutionary interventions, have gradually become less effective as microorganisms develop mechanisms to resist treatment [2]. AMR is among the most significant global public health threats, with an estimated 1.27 million deaths directly attributed to it and up to 4.95 million deaths indirectly associated with resistant infections in 2019 [3]. AMR occurs across all countries and income levels, with the largest burden experienced in low- and middle-income countries, which are disproportionately affected by the underlying drivers and consequences of antimicrobial resistance [3]. As a result, infections become harder to treat, and routine clinical care increasingly carries higher risks.

This burden is disproportionately borne by low- and middle-income countries, reinforcing existing health and socioeconomic inequalities. The mortality associated with AMR is now comparable to, or exceeds, that of HIV/AIDS and malaria combined, highlighting the scale and urgency of the problem [4]. In addition to increased mortality, AMR weakens medical advances, including surgery, cesarean delivery, oncology, neonatology, and intensive care, thereby jeopardizing decades of progress in population health and socioeconomic development [5].

AMR epidemiology is multifaceted, arising from the interaction of biological evolution, human behaviour, healthcare practices, agriculture, and environmental contamination [6]. Although microbial adaptation is a natural evolutionary process, human activities substantially accelerate this process [7]. Inappropriate antibiotic use, self-medication, and incomplete treatment courses create selective pressure that enables resistant strains to proliferate [8]. Similarly, widespread antimicrobial use in livestock and aquaculture for growth promotion and disease prevention increases resistance reservoirs in food chains and natural ecosystems [9]. Environmental dissemination through hospital effluents, pharmaceutical waste, and agricultural runoff further spreads resistant genes into soil, surface water, and wildlife [10]. Accordingly, AMR should be understood not only as a biomedical issue but also as a broader ecological and societal challenge shaped by systemic and structural factors [11].

The geographical and socioeconomic distribution of AMR reflects pronounced global inequities [12]. Low- and middle-income countries are disproportionately affected due to limited healthcare infrastructure, weak regulatory enforcement, and unrestricted access to antibiotics [13]. In contrast, high-income countries face AMR challenges largely related to hospital outbreaks and the effects of globalization, including medical tourism and international trade [14]. Regional variations in surveillance capacity and stewardship implementation are evident, such as the north-south gradient observed in Europe and the high prevalence reported in South Asia and Sub-Saharan Africa [15]. Nevertheless, robust and comparable global estimates remain limited, as many regions lack consistent longitudinal surveillance data [16]. These evidence gaps hinder accurate burden estimation and the development of targeted interventions [17].

**Figure 1:**
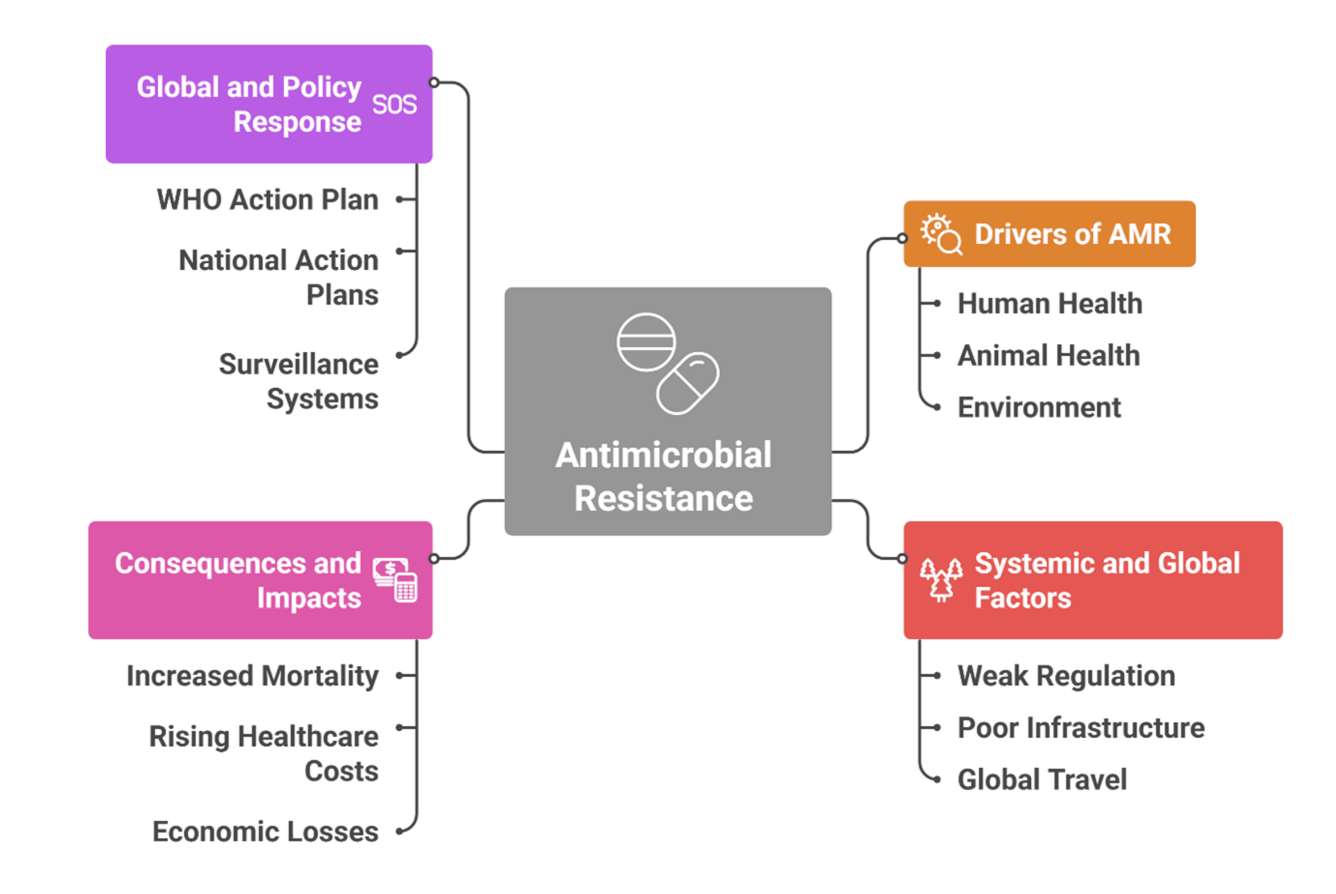
Key factors and responses influencing antimicrobial resistance (AMR) Created by authors using Napkin AI software

Recognizing these interconnections, the One Health approach has become central to understanding and addressing AMR [18]. This framework integrates human, animal, and environmental health systems to clarify transmission pathways and support coordinated responses [19]. International surveillance programmes, including the WHO Global Antimicrobial Resistance and Use Surveillance System (GLASS), the European Antimicrobial Resistance Surveillance Network (EARS-Net), and regional initiatives such as CAESAR and ReLAVRA, have strengthened global monitoring efforts [20]. However, persistent gaps remain in data harmonization, laboratory quality assurance, and integration across human and veterinary sectors, particularly in resource-limited settings [21]. Environmental surveillance is especially underdeveloped and underfunded, leaving major gaps in understanding resistance ecology [22]. These limitations highlight the need for sustained capacity-building, standardization, and financing to operationalize One Health approaches in practice. The key factors and responses influencing AMR are illustrated in Figure 1.

The economic consequences of AMR are substantial. Resistant infections increase healthcare costs through longer hospital stays, greater use of intensive care, and reliance on costly last-resort antibiotics. According to the WHO, uncontrolled AMR could reduce global GDP by up to 3.8% by 2050, with low-income economies bearing the greatest impact and facing heightened poverty risks [23]. However, empirical evidence on indirect social and economic costs-such as productivity loss and household financial strain-remains limited, relying largely on modelling studies. This lack of real-world cost-effectiveness data complicates policymaking and investment decisions.

International policy responses have sought to address AMR through frameworks such as the WHO Global Action Plan on Antimicrobial Resistance (2015), which emphasizes awareness, surveillance, optimized antimicrobial use, and sustained innovation. By 2023, more than 150 countries had developed national AMR action plans aligned with these principles [24]. Nevertheless, implementation remains uneven, with many plans lacking measurable indicators, cross-sectoral coordination, or sustainable financing. Behavioural, cultural, and institutional barriers continue to hinder effective stewardship and infection prevention. These shortcomings suggest that technical solutions alone are insufficient without long-term political commitment and societal engagement.

Despite the growing AMR literature, critical knowledge gaps persist. Limited evidence exists on how governance structures, socio-behavioural factors, and health system resilience interact to shape resistance patterns. Comparative evaluations of the long-term sustainability and impact of national AMR programmes within a One Health framework are scarce. Environmental drivers, including pharmaceutical pollution and climate variability, also remain underexplored. Addressing these gaps will require integrative, interdisciplinary research spanning epidemiology, economics, behavioural science, and policy analysis.

Objectives of the review

The overarching goal of this review is to provide an integrated One Health perspective on antimicrobial resistance (AMR) by synthesizing global epidemiological, environmental, socioeconomic, and governance evidence. The review maps global and regional resistance patterns, examines key drivers across human, animal, and environmental systems, and evaluates the effectiveness of current surveillance and policy responses. Its novel contribution lies in linking epidemiological trends with economic and governance challenges to identify implementation gaps and priority areas for coordinated, cross-sectoral action, thereby supporting more effective and sustainable AMR control strategies.

## Review

Methodological considerations

Study Design and Analytical Framework

A narrative synthesis was employed to integrate multidisciplinary evidence on the global epidemiology of antimicrobial resistance (AMR) in humans, animals, and the environment, aligned with a One Health perspective. This approach was selected to enable a broad, systems-level interpretation of AMR trends, drivers, and responses across sectors where quantitative synthesis was not feasible. Synthesis was guided by a systems-based analytical framework using epidemiological principles of distribution, determinants, and transmission dynamics, allowing for a comprehensive interpretation of AMR patterns without overemphasizing individual data sources. Given the narrative design, the objective was to enhance conceptual integration and transparency rather than achieve full procedural reproducibility characteristic of systematic reviews.

Search Strategy and Data Sources

Systematic reviews, meta-analyses, peer-reviewed original studies, and policy documents published between 2015 and 2025 were identified through structured electronic searches of PubMed, Scopus, Web of Science, and Google Scholar. Search terms were combined using Boolean operators and included “antimicrobial resistance,” “antibiotic resistance,” “epidemiology,” “surveillance,” “prevalence,” and “determinants.” Additional evidence was obtained from publicly available international and national surveillance reports, official health agency publications, and reference lists of eligible articles to ensure comprehensive coverage of relevant literature. Representative search combinations included (“antimicrobial resistance” AND epidemiology), (“antibiotic resistance” AND surveillance), and (“AMR” AND One Health).

Eligibility Criteria and Study Selection

Studies were included if they presented quantitative or qualitative data on AMR burden, trends, determinants, surveillance, or policy responses across human, animal, or environmental domains. Commentaries, duplicate records, and publications lacking clear methodological descriptions were excluded. Study selection prioritized relevance to global or regional AMR patterns and alignment with the One Health framework. Screening was conducted in two stages, beginning with title and abstract review, followed by full-text assessment of potentially eligible articles. Study selection was performed by a single reviewer, with eligibility decisions guided by predefined inclusion and exclusion criteria.

Quality Appraisal and Limitations

Each included source was assessed for methodological rigour, representativeness, and potential sources of bias. No formal risk-of-bias scoring tool was applied; instead, study quality was appraised narratively, consistent with the aims of a narrative review. Variability in laboratory practices, data completeness, surveillance coverage, and reporting standards across regions was recognized as an inherent limitation of the available evidence. These constraints were considered during synthesis to avoid overinterpretation of heterogeneous findings.

Global burden and distribution of antimicrobial resistance

Global Mortality and Health System Impact

Antimicrobial resistance (AMR) is a major global health concern, causing approximately 1.27 million deaths in 2019 and contributing to nearly 5 million deaths associated with resistant infections [4]. Its burden is comparable to that of HIV/AIDS and malaria and disproportionately affects populations in Sub-Saharan Africa and South Asia, particularly children and vulnerable adults [23]. Beyond mortality, AMR increases morbidity, prolongs illness duration, elevates healthcare costs, and reduces quality of life, placing sustained pressure on health systems worldwide [24].

Priority Pathogens and Emerging Resistance Threats

The World Health Organization classifies resistant pathogens into critical, high, and medium priority groups based on their public health threat [25]. Critical-priority pathogens include carbapenem-resistant Acinetobacter baumannii, Pseudomonas aeruginosa, and Enterobacteriaceae. High- and medium-priority organisms include methicillin-resistant Staphylococcus aureus (MRSA), vancomycin-resistant Enterococcus faecium, penicillin-non-susceptible Streptococcus pneumoniae, and ampicillin-resistant Haemophilus influenzae [26,27]. Resistance has also emerged in pathogens such as Mycobacterium tuberculosis, Candida auris, and Aspergillus fumigatus, further complicating treatment and global disease control efforts [6].

Healthcare- and Community-Associated Resistance

Healthcare-associated infections remain a major contributor to the AMR burden. S. aureus, P. aeruginosa, E. coli, K. pneumoniae, and Enterococcus species frequently exhibit methicillin, carbapenem, ESBL, and vancomycin resistance in hospital settings [11,28]. Concurrently, community-associated resistance is increasing, with resistant E. coli, MRSA, and drug-resistant Streptococcus pneumoniae increasingly detected outside healthcare environments [29]. Foodborne and sexually transmitted pathogens, including Salmonella, Campylobacter, Shigella, and Neisseria gonorrhoeae, are also demonstrating rising resistance, often associated with antimicrobial use in agriculture [30].

Geographic Variation and Global Inequities

Rather than reiterating regional comparisons described elsewhere, this section highlights that persistent geographic variation in AMR reflects underlying socioeconomic, infrastructural, and regulatory disparities. These inequities reinforce the need for coordinated global surveillance, equitable resource allocation, and integrated interventions within a unified One Health framework [31].

Determinants and risk factors for antimicrobial resistance

Human Health-Related Drivers

The problem of antimicrobial resistance (AMR) arises from a complex interaction of biological, behavioural, environmental, and systemic factors, with the overuse and misuse of antimicrobials in human medicine representing a primary driver [32]. Inappropriate prescribing, particularly for viral infections such as upper respiratory tract illnesses, bronchitis, and influenza, remains common, with an estimated 30-50% of outpatient antibiotic use considered unnecessary [33]. These practices are often driven by diagnostic uncertainty, patient expectations, and limited access to rapid diagnostic testing [5]. Errors in antibiotic selection, dosing, and treatment duration, along with self-medication and premature treatment discontinuation, further accelerate resistance development [10]. System-level influences, including fee-for-service healthcare models, pharmaceutical marketing, and weak antimicrobial stewardship programmes, exacerbate these patterns [12].

Animal Health and Agricultural Drivers

In the agricultural sector, antimicrobial use in livestock for growth promotion and disease prevention often exceeds that in human medicine [34]. Human exposure occurs through direct contact with animals, consumption of contaminated food products, and environmental pathways [7]. The use of medically important antimicrobials such as fluoroquinolones and macrolides in food-producing animals has facilitated the transfer of resistance genes to human pathogens, including the global spread of colistin resistance mediated by mcr genes [18]. Similar antimicrobial practices in aquaculture contribute to resistance dissemination through contamination of water systems [20].

Healthcare-Associated Transmission

Healthcare facilities represent critical environments for the amplification and spread of resistant organisms [15]. Patients receiving broad-spectrum antibiotics, undergoing invasive procedures, or exposed to inadequate infection prevention measures are particularly vulnerable to colonization and infection with resistant opportunistic pathogens, including Clostridioides difficile and vancomycin-resistant Enterococcus [8]. Long-term care facilities, characterized by frequent antimicrobial use and limited resources, act as reservoirs for resistant organisms that may be reintroduced into acute care settings through patient transfers [9].

Environmental Contamination and Ecological Spread

Environmental contamination constitutes another major dimension of AMR transmission. Hospital effluents, pharmaceutical manufacturing waste, and agricultural runoff introduce antimicrobial residues and resistant bacteria into soil and water systems [35]. Improper disposal practices during pharmaceutical production release high concentrations of antimicrobials into the environment, intensifying selective pressure on microbial populations [6]. Companion animals and wildlife further contribute to dissemination through fecal contamination and migratory pathways [11].

Globalization and Population Mobility

Globalization accelerates the international spread of AMR through increased travel, trade, and migration [13]. Medical tourists and international travellers may acquire resistant organisms, such as ESBL-producing Enterobacteriaceae, in high-prevalence regions and subsequently introduce them into low-prevalence settings [36]. The global movement of people, animals, and goods enables rapid cross-border transmission of resistance genes [4].

Socioeconomic and Governance Factors

**Figure 2:**
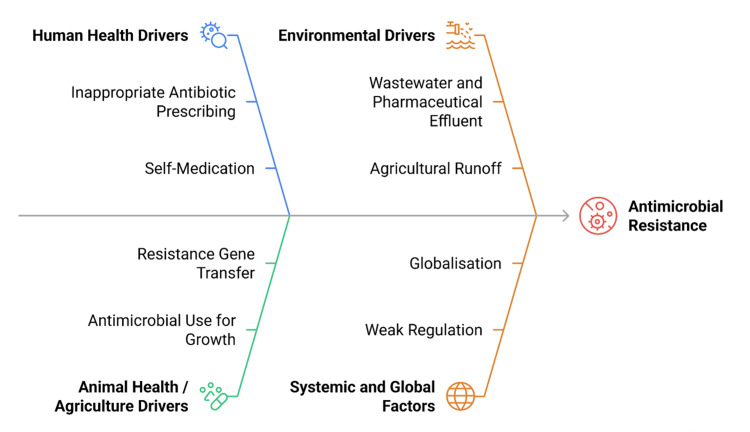
Key human, animal, environmental, and systemic drivers leading to antimicrobial resistance (AMR) Created by authors using Napkin AI software

Broader socioeconomic and governance conditions play a decisive role in shaping AMR dynamics [2]. Limited access to healthcare, poverty, malnutrition, and population overcrowding increase susceptibility to infection and inappropriate antibiotic use [14]. Weak regulatory frameworks allow unregulated antimicrobial sales and the circulation of substandard or counterfeit medicines, particularly in low-income settings [1]. Together, these interconnected human, animal, environmental, and systemic drivers underscore the necessity of a One Health approach, emphasizing coordinated, multisectoral responses to curb the global rise of AMR. Figure 2 summarises the key determinants across these domains.

Surveillance and monitoring of antimicrobial resistance

Role of Surveillance in AMR Control

In order to understand, monitor, and control antimicrobial resistance (AMR), effective surveillance is a prerequisite [37]. Vigorous surveillance systems facilitate the monitoring of resistance patterns, identification of emerging threats, and evaluation of intervention effectiveness [14]. Surveillance data also underpin empiric treatment guidelines, antimicrobial stewardship programmes, and national and international policy development, reinforcing their central role in AMR containment strategies [12].

Global, Regional, and National Surveillance Systems

Over the past decade, global, regional, and national AMR surveillance programmes have expanded substantially [38]. The WHO Global Antimicrobial Resistance and Use Surveillance System (GLASS) provides a globally standardized framework that enables cross-country comparability, although participation and data completeness remain variable across regions [16]. Regional networks, including the European Antimicrobial Resistance Surveillance Network (EARS-Net), the Central Asian and Eastern European Surveillance of Antimicrobial Resistance (CAESAR), and the Latin American Antimicrobial Resistance Surveillance Network (ReLAVRA), offer higher-resolution epidemiological data tailored to regional priorities, though their findings are less generalizable beyond their geographic scope [39].

At the national level, systems such as the CDC’s National Healthcare Safety Network, ESPAUR in England, and Canada’s CARSS provide detailed insights into local resistance patterns and antimicrobial consumption trends, supporting targeted stewardship and policy actions. However, methodological heterogeneity across national systems limits direct international comparisons [18]. Pathogen-specific programmes, including the WHO Global Tuberculosis Programme and the Gonococcal Antimicrobial Surveillance Programme (GASP), provide sustained and in-depth monitoring of priority pathogens but do not capture the broader AMR landscape [15].

Surveillance Methodologies and Data Sources

Surveillance approaches differ according to their objectives and available resources [40]. Laboratory-based surveillance remains the most widely used method and is efficient for detecting resistance trends in severe and hospital-based infections, although it tends to underrepresent community-level disease burden [19]. Population-based surveillance provides more accurate incidence estimates and improved representativeness but is resource-intensive and challenging to sustain, particularly in low-resource settings [17].

Molecular surveillance, including whole-genome sequencing and metagenomics, offers high-resolution insights into resistance mechanisms and transmission pathways, enabling real-time outbreak detection; however, high costs and technical requirements limit widespread implementation [11,20]. Monitoring antimicrobial consumption, particularly through the WHO AWaRe classification, complements resistance surveillance by identifying misuse patterns and informing stewardship interventions [13].

Challenges and Gaps in Surveillance

Despite these advances, AMR surveillance systems face persistent challenges, especially in low- and middle-income countries [41]. Inadequate laboratory capacity, inconsistent testing standards, and limited external quality assurance undermine data reliability and comparability [42]. Fragmentation across human, animal, and environmental sectors further restricts integrated analysis, while underdeveloped electronic data systems and limited data sharing impede timely decision-making [14,18]. Community-based and veterinary surveillance remain particularly underdeveloped, creating substantial blind spots in the global understanding of resistance dynamics [16].

Future Directions and Integration

Looking ahead, the relative strengths of existing surveillance systems highlight the need for integration rather than replacement. Advances in rapid diagnostics, point-of-care testing, and electronic reporting can improve data timeliness and coverage [13]. Combining standardized global platforms such as GLASS with regionally and nationally tailored systems may achieve both international comparability and local relevance [20]. Emerging applications of artificial intelligence and machine learning offer additional opportunities to detect trends, predict resistance emergence, and prioritize interventions. Continued investment in laboratory capacity, methodological harmonization, and cross-sectoral data sharing remains essential to building an effective, equitable, and One Health-oriented AMR surveillance infrastructure.

Public health interventions and control strategies

Integrated Strategies for AMR Control

Addressing antimicrobial resistance (AMR) requires coordinated, evidence-based interventions across antimicrobial use, infection prevention, diagnostics, research, and public awareness [43]. When effectively implemented, these measures reduce inappropriate antimicrobial use, limit transmission of resistant organisms, and help preserve the effectiveness of existing therapies [27].

Antimicrobial Stewardship Programmes

Antimicrobial stewardship programmes (ASPs) are central to AMR control [44]. These structured initiatives promote the optimal use of antimicrobials to improve patient outcomes while minimizing resistance development [21]. Core elements of effective ASPs include institutional leadership commitment, accountability through designated stewardship leaders, and expert oversight by infectious disease specialists and pharmacists. Key stewardship activities include prospective audit and feedback, formulary restriction, and development of evidence-based local treatment guidelines [19].

Regular monitoring of antimicrobial consumption and resistance trends enhances accountability, while targeted education strengthens appropriate prescribing practices [23]. Meta-analyses demonstrate that stewardship interventions can reduce antimicrobial use by 10-40%, lower rates of infections caused by resistant organisms, and decrease healthcare costs without compromising patient safety [26]. Nevertheless, implementation barriers persist, including limited resources, diagnostic delays, and prescriber resistance [22].

Infection Prevention and Control

Infection prevention and control (IPC) measures are critical for limiting the transmission of resistant organisms in healthcare and community settings [45]. IPC relies on standard precautions such as hand hygiene, appropriate use of personal protective equipment, safe injection practices, and environmental cleaning [24]. Transmission-based precautions, including contact, droplet, and airborne measures, are applied based on pathogen-specific risks [25].

Early identification of carriers through surveillance and targeted screening of high-risk patients supports timely containment [28]. Enhanced environmental disinfection, including sporicidal agents for Clostridioides difficile and advanced technologies such as ultraviolet radiation and hydrogen peroxide vapour systems, further reduces healthcare-associated transmission [20]. Community-level IPC strategies, safe food handling, sanitation, and vaccination-also contribute to lowering infection risk [30].

Vaccination as a Preventive Strategy

Vaccination plays a crucial role in AMR prevention by reducing infection incidence and subsequent antibiotic exposure [29]. Herd immunity resulting from pneumococcal conjugate vaccines (PCVs) has led to significant reductions in multidrug-resistant Streptococcus pneumoniae infections [46]. Similarly, Haemophilus influenzae type b (Hib) vaccination has nearly eliminated invasive Hib disease, while influenza vaccination prevents viral infections that often lead to unnecessary antibiotic prescribing [27]. Typhoid conjugate vaccines reduce the burden of drug-resistant Salmonella Typhi [23]. Vaccines targeting pathogens such as Escherichia coli, Klebsiella pneumoniae, Staphylococcus aureus, and Shigella are under development, although cost and implementation challenges remain substantial, particularly in low-income settings [25].

Diagnostics and Targeted Therapy

Effective stewardship and IPC are reinforced by timely and accurate diagnostics [44]. Conventional culture-based methods remain reliable but are often time-consuming [24]. Rapid diagnostic technologies, including polymerase chain reaction (PCR) assays and matrix-assisted laser desorption ionization time-of-flight mass spectrometry (MALDI-TOF MS), enable faster pathogen identification and resistance detection, facilitating early targeted therapy [47].

Biomarkers such as C-reactive protein and procalcitonin help distinguish bacterial from viral infections, reducing inappropriate antibiotic use in outpatient settings [22]. Although cost and infrastructure constraints limit widespread adoption, settings that integrate rapid diagnostics with stewardship programmes demonstrate improved therapeutic precision and outcomes [20].

Research, Innovation, and Public Engagement

Continued research and innovation are essential to address the stagnation in antibiotic development [43]. Scientific, regulatory, and economic barriers have limited the introduction of new antimicrobials, necessitating incentives such as public-private partnerships, market entry rewards, and regulatory support mechanisms [26]. Alternative approaches, including bacteriophage therapy, antimicrobial peptides, monoclonal antibodies, and microbiome-based interventions, are under active investigation [21].

Public education and awareness campaigns are also critical for promoting responsible antimicrobial use [45]. Campaigns delivered through mass media, healthcare professionals, and educational institutions have been shown to reduce inappropriate antibiotic demand and improve adherence to prescribed treatments [19]. Sustained multimedia interventions have achieved community-level reductions in antibiotic use of up to 15% [46]. Table 1 summarises the major intervention domains, strategies, outcomes, and supporting evidence for AMR control.

**Table 1:** Key interventions and strategies for mitigating antimicrobial resistance PPE: Personal protective equipment; MALDI-TOF-MS: Matrix-assisted laser desorption ionization time-of-flight mass spectrometry; CRP: C-reactive protein.

Intervention Area	Primary Focus	Core Strategies / Actions	Key Outcomes and Evidence	References
Antimicrobial Stewardship Programs (ASPs)	Optimizing antimicrobial use and improving clinical outcomes	Institutional leadership and accountability; expert oversight by infectious disease specialists and pharmacists; formulary restrictions; prospective audit and feedback; education on rational prescribing; monitoring antimicrobial use and resistance trends	Reduces antimicrobial use by 10–40%; lowers infection rates from resistant organisms; decreases treatment costs; maintains patient safety	[33]
Infection Prevention and Control (IPC)	Preventing transmission of resistant organisms in healthcare and community settings	Standard precautions (hand hygiene, PPE, safe injections, disinfection); additional precautions (contact, droplet, airborne); active surveillance; environmental cleaning; advanced decontamination (UV, hydrogen peroxide vapor); community-level sanitation and food safety	Decreases infection spread; improves hospital hygiene; enhances outbreak containment; strengthens community resilience	[28]
Vaccination	Reducing infection incidence and antibiotic use	Immunization against Streptococcus pneumoniae, H. influenzae type b, influenza, and typhoid; development of vaccines against E. coli, K. pneumoniae, S. aureus, and Shigella	Prevents bacterial and viral infections; decreases antimicrobial prescriptions; provides herd protection; reduces resistant infections	[12]
Diagnostics	Enabling rapid detection and targeted therapy	Rapid diagnostic tools (PCR, MALDI-TOF MS); point-of-care biomarker tests (CRP, procalcitonin); integration of diagnostics into stewardship programs	Improves therapeutic precision; shortens time to appropriate treatment; reduces unnecessary antibiotic use; is cost-effective in the long term.	[20]
Research and Innovation	Stimulating new treatment and control options	Incentives for antimicrobial R&D (public–private partnerships, market entry rewards); exploration of bacteriophage therapy, antimicrobial peptides, monoclonal antibodies, and microbiome approaches	Promotes drug innovation; diversifies therapeutic options; addresses stagnation in antibiotic discovery	[45]
Public Awareness and Education	Promoting responsible antimicrobial behavior	Mass media campaigns; healthcare engagement; community education; school programs	Increases awareness; reduces unnecessary antibiotic demand; promotes adherence; decreases community antibiotic use by up to 15%	[47]

Economic Impact and Cost-Effectiveness

Antibiotic-resistant infections require costlier drugs, extended hospitalisation, and intensive care, which considerably increases the healthcare spending [47]. The treatment of resistant infections may include intravenous therapy over oral therapy, prolonged periods, and complicated management of complications in patients [23]. The healthcare system in the United States is estimated to pay more than 4.6 billion US dollars a year due to antibiotic-resistant infections, and per-case costs are as high as 2 times higher than those of infections caused by susceptible pathogens [28]. In Europe, the aggregate annual cost of AMR, both in healthcare expenditure and loss of productivity, is over one and a half [31]. Worldwide, premature mortality and long-term disability are also causes of high productivity loss, and caregivers also tend to have their income interrupted because of long-term sickness in relatives [36]. According to the projection of the World Bank, the extreme AMR scenarios would decrease global GDP by 1.1-3.8% by 2050, and the greatest amounts of decrease will be experienced by low- and middle-income nations [48].

The economic justification of AMR interventions is always high according to the cost-effectiveness analyses [22]. Both the hospital-based antimicrobial stewardship programs are also cost-effective and may lead to a payback period of one to two years due to a decrease in drug spending, decreased hospitalization, and avoidance of expensive complications [25]. Such measures as hand hygiene programs and decolonisation measures are also cost-effective in terms of infections, and universal interventions have been particularly effective in high-prevalence environments [35]. Vaccinations such as pneumococcal conjugate and influenza vaccines save a lot of costs as they help to avoid infections that would otherwise require the use of antibiotics and hospitalisation [49]. On the same note, a quick diagnostic test may enhance better prescription and reduce the cost of treatment when used in combination with stewardship methods [27].

Regardless of the obvious economic indicators, the efforts of AMR response are chronically underfunded [30]. The global Commission on Antimicrobial Resistance has approximated that the global effort to enhance surveillance, stewardship, infection prevention, research, and capacity-building efforts in low-resource environments would need approximately $40 billion in a decade [50]. Nevertheless, the present investments are miles below this target [24]. Both health and economic impacts of AMR can thus be reduced through strategic resource allocation and long-term funding [40].

Policy and Governance Approaches

Policy and governance are critical to organising the global, national, and sectoral activities in antimicrobial resistance (AMR) [42]. In 2015, the World Health Organisation (WHO) introduced the Global Action Plan on AMR, and all member states were asked to prepare national action plans that would be consistent with five strategic objectives, including raising awareness, enhancing surveillance, lowering the incidence of infections, optimising the use of antimicrobials, and ensuring sustainable investment [15]. Over 150 countries had developed national AMR action plans by 2023, but implementation is still uneven [33]. Countries with high income tend to have more robust systems of governance and the allocation of resources, and those with low and middle income (LMICs) are often affected by the lack of capacity, poor coordination, and resources [9]. Long-term political commitment, multisectoral participation, and the existence of well-specified accountability mechanisms are required to ensure effective implementation [25].

The AMR control involves regulatory frameworks [46]. Prescription-only policy plays a critical role in human health by limiting the sale of over-the-counter antibiotics, but in most areas, it is not enforced [11]. The quality assurance mechanisms should protect against fake and bad quality drugs, whereas regulations of pharmaceutical marketing are required to ensure that inappropriate prescribing is avoided [30]. Bans on the use of antimicrobials to stimulate growth in food-producing animals that have been well enforced in the European Union and the United States serve as an example in the veterinary sector [38]. There is a need for veterinary supervision, regulations on feed additives, and monitoring of the antimicrobial use to promote responsible behaviour [20]. Environmental control, which is less advanced, is also becoming accepted as crucial, especially in restricting the discharge of antimicrobials by pharmaceutical production and agricultural wastes [27].

International collaboration is a necessity, as AMR is transboundary in nature [44]. The One Health approach to governance is the three-part cooperation between the WHO, Food and Agriculture Organisation (FAO), and World Organisation for Animal Health (OIE), in which the three areas of human, animal, and environmental health are integrated [32]. These organs are responsible for coordinating surveillance, formulating international principles, and aiding technical capacity-building in the member states [12]. The high-level meeting on AMR at the United Nations General Assembly in 2016 was a step towards taking the issue to the world political agenda, where countries pledged to act in concert [19]. Agreements set by international organisations like the Codex Alimentarius and the agreements of the World Trade Organisation also have an effect on food safety and the use of antimicrobial standards [26].

Nevertheless, the global governance is characterised by some ongoing problems, such as resource allocation inequities, the issues of national sovereignty, the problem of unequal distribution of data, and inadequate long-term funding [48]. The solution to these gaps needs to be enhanced accountability, equal investment, and long-lasting international solidarity [37]. The major policy domains, actions, and implementation gaps in addressing AMR are summarised in Table 2.

**Table 2:** Policy frameworks and governance mechanisms addressing antimicrobial resistance

Policy Domain	Key Focus Areas	Major Actions and Frameworks	Challenges and Gaps	References
Global Policy and Coordination	Strengthening collective international response to AMR	WHO Global Action Plan on AMR (2015); five strategic objectives—awareness, surveillance, infection reduction, optimal use, and investment; over 150 national action plans developed by 2023	Uneven implementation, limited monitoring, and resource and governance disparities between countries	[4]
National Governance and Regulation	Enforcing responsible antimicrobial use and ensuring drug quality	Prescription-only antibiotic policies; pharmaceutical marketing controls; quality assurance systems; bans on growth promoters in livestock (EU, USA); veterinary oversight and feed regulations	Weak law enforcement, unregulated over-the-counter sales, counterfeit drugs, and poor intersectoral coordination	[46]
Environmental Regulation	Controlling antimicrobial pollution from human, industrial, and agricultural sources	Inclusion of environmental discharge limits, waste management, and monitoring of pharmaceutical industries, regulation of agricultural runoff, and effluents	Weak environmental governance; limited surveillance capacity; lack of standardized metrics	[35]
International Collaboration (One Health Approach)	Integrating human, animal, and environmental health under global frameworks	Tripartite collaboration (WHO–FAO–WOAH); Codex Alimentarius standards; WTO guidelines; UN General Assembly high-level meeting on AMR (2016)	Inequitable resource distribution, sovereignty issues, inconsistent data sharing, and insufficient long-term financing	[45]

One Health Approach to Antimicrobial Resistance

The One Health strategy acknowledges the interdependence of human, animal, and environmental health and the need to work together across the three sectors to reduce antimicrobial resistance (AMR) [28] in an effective manner. Resistance is not something that develops in a vacuum; it comes into existence and propagates via interdependent mechanisms that are affected by human actions, farming techniques, and pollution of the environment [33]. It is thus critical to have a single framework to comprehend the dynamics of resistance transmission and to develop coordinated intervention [40].

A One Health strategy must be implemented in a well-organized coordination of ministries of health, agriculture, environment, and others [19]. National One Health committees or intersectoral platforms have been set up in many countries as a way of data sharing, combining plans, and harmonizing policies [22]. Human, veterinary, and environmental data can be connected to track the ecology of the resistance, which will give a comprehensive picture of the problem and allow for revealing the pathways of its spread early and identifying the new threats [45]. The uniformity of methods used in the sectors boosts comparability and facilitates the responsive actions [27]. The cross-sectoral teams conduct joint risk assessments to assist in prioritizing interventions that are based on the aggregate evaluation of risks across all domains [48].

Communication and capacity building are core values of the One Health model [35]. Medical, veterinary, and environmental training Collaborative training of medical, veterinary, and environmental professionals promotes mutual understanding and skill sharing [12]. Cross-disciplinary studies of resistance in food webs, water, and wild animals produce important information on the mechanisms of transmission and response to intervention [23]. Nevertheless, most of the practical aspects of One Health approaches still pose challenges, such as institutional silos, constraints in the financing of resources, and variation in data systems and regulatory cultures [44]. Fiscal factors and weak political will, in addition to the lack of confidence in the cost-effectiveness, further hinder the progress [17].

Regardless of such obstacles, the One Health framework is a pillar of sustainable AMR management [42]. It can integrate surveillance, governance, research, and capacity development aspects across sectors and hence fits the international call for a coordinated and multisectoral response to curb resistance [49]. Further investment, interdisciplinary partnerships, and policy alignment are crucial in the implementation of the concept of One Health and making significant gains in curbing the global AMR burden [38].

Critical Gaps, Challenges, and Future Directions

Although significant progress has been made on antimicrobial resistance (AMR), there are still several critical issues. One of the main issues is the trade-off between access and overuse of antimicrobials, making sure that there is fair access to quality-assured medicines and eliminating the wrong use of antimicrobials, which contributes to resistance. The low-income countries have been known to have two burdens of inaccessibility and uncontrolled distribution. There are still gaps in the research on the long-term sustainability of the stewardship and infection prevention programs, the role of environmental contamination in resistance, and the effectiveness of One Health and sector-specific approaches. Barriers to implementation are also obstructed by political, financial, and governance barriers, and national fragmentary responses, combined with poor international coordination, bring progress at a crawl.

The new course of action should also be integrated and multisectoral. There is a need to enhance surveillance measures, especially in resource-constrained environments, to monitor trends and to lead resourceful interventions. Both infection and antimicrobial use and transmission can be reduced through the expansion of stewardship and infection control programs to all levels of healthcare. There needs to be long-term investment in the research and development of new antimicrobials, diagnostics, and vaccines, which are accompanied by equal access throughout the world. It is important to operationalize the One Health principles based on the coordinated policy and surveillance, engage the population in behavioural change, and guarantee long-term funding. To save the growing menace of AMR, worldwide collaboration, collective responsibility, and long-term political dedication are ultimately needed to maintain antimicrobial efficacy.

## Conclusions

AMR is a growing worldwide health crisis that disregards the efficacy of contemporary medicine and the sustainability of development. As highlighted in this review, AMR is caused by a complex interaction of biological, behavioural, and systemic factors in human, animal, and environmental space. Although the world has made progress in its surveillance, stewardship, and prevention of infection, there are deep resource, capacity, and policy inequality gaps, especially in the low- and middle-income nations. These inequalities require concerted investment in global surveillance systems, sensible use of antimicrobials, innovative treatment, and vaccine development. The One Health model is a paradigm that provides a comprehensive approach to the transmission of resistance, as well as coordination of cross-sectoral responses. Enhanced leadership, citizen participation, and fair funding are critical to the continuation of developments. Finally, the need to curb AMR will involve turning commitment into action at a global level to be concrete and sustained in order to maintain antimicrobial action, safeguard future generations, and health system resilience across the borders of the world. The achievements of modern medicine are at risk of being washed away in an ever more hostile bacterial world in the absence of such concerted effort.
